# Abundant Human DNA Contamination Identified in Non-Primate Genome Databases

**DOI:** 10.1371/journal.pone.0016410

**Published:** 2011-02-16

**Authors:** Mark S. Longo, Michael J. O'Neill, Rachel J. O'Neill

**Affiliations:** Department of Molecular and Cell Biology, University of Connecticut, Storrs, Connecticut, United States of America; The University of Maryland, United States of America

## Abstract

During routine screens of the NCBI databases using human repetitive elements we discovered an unlikely level of nucleotide identity across a broad range of phyla. To ascertain whether databases containing DNA sequences, genome assemblies and trace archive reads were contaminated with human sequences, we performed an in depth search for sequences of human origin in non-human species. Using a primate specific SINE, AluY, we screened 2,749 non-primate public databases from NCBI, Ensembl, JGI, and UCSC and have found 492 to be contaminated with human sequence. These represent species ranging from bacteria (*B. cereus*) to plants (*Z. mays*) to fish (*D. rerio*) with examples found from most phyla. The identification of such extensive contamination of human sequence across databases and sequence types warrants caution among the sequencing community in future sequencing efforts, such as human re-sequencing. We discuss issues this may raise as well as present data that gives insight as to how this may be occurring.

## Introduction

The danger in the propagation of errors in scientific discourse has been demonstrated in cases of both scientific fraud as well as incorrectly described or referenced experiments in reviews [1], [2]. As sequencing technologies become more robust, efficient and affordable, the number of genome sequencing projects is increasing exponentially. While human DNA contamination has been a concern for both ancient [3], [4] and forensic samples [5], there has been no attempt to systematically identify or quantify human contamination in public genome databases since the advent of next generation sequencing or genome assemblies. Contamination of non-primate databases with human sequence confounds comparative analyses, gene annotation, and regulatory network analyses among many others. Moreover, the identification of such contamination in non-primate databases would indicate that more robust pre-sequencing pipelines should be established to limit cross contamination among human genome sequences. We set out to determine whether, and to what extent, human DNA contamination could be identified in non-primate genome assemblies and other DNA databases. We used the primate specific repeat AluY as a query sequence to identify instances of human contamination in all the non-primate NCBI trace archives and genome assemblies, the University of California Santa Cruz assemblies (UCSC), Ensembl and the Joint Genome Institute databases (JGI).

## Results

AluY belongs to the primate specific class of short nucleotide elements (SINEs) and was chosen because of its abundance in the human genome (over 1 million copies [6]), its specificity to the primate lineage [6], [7], and its length (282 bp). This short length was a critical variable for analyzing trace archive reads as they are often entries of 1 kb or less. This small query sequence provided sufficient adjacent, unique sequence to allow for unambiguous mapping of Alu-containing DNA to a single locus in the human genome. AluY sequences were identified in non-human databases using the BLASTN alignment algorithm (BLAT for UCSC). Sequences with nucleotide identity of >80% were collected and further analyzed. Our low initial screening stringency allowed the identification of other members of the Alu family, although it limited our approach to non-primate genomes. Sequences were considered human contamination if they were >98% identical to human sequence and mapped to a single, unique locus in the human genome (NCBI Build 37.1).

**Figure 1:**
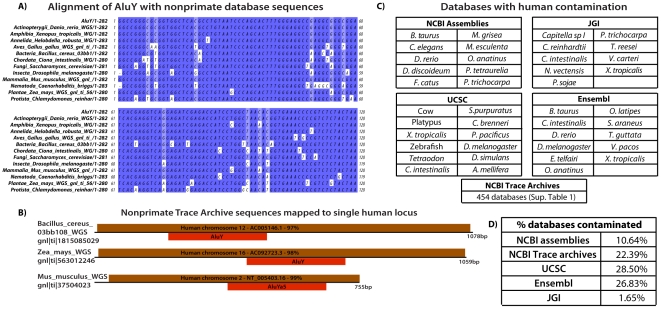
Human sequences found in non-primate databases. A) Representative Clustal alignment of NCBI non-primate trace archive reads to a consensus primate specific AluY. B) Representative NCBI non-primate trace archive reads mapped to single human locus. C) Summary of non-primate databases contaminated with human sequence. D) Percent of public databases identified as contaminated.

**Figure 2:**
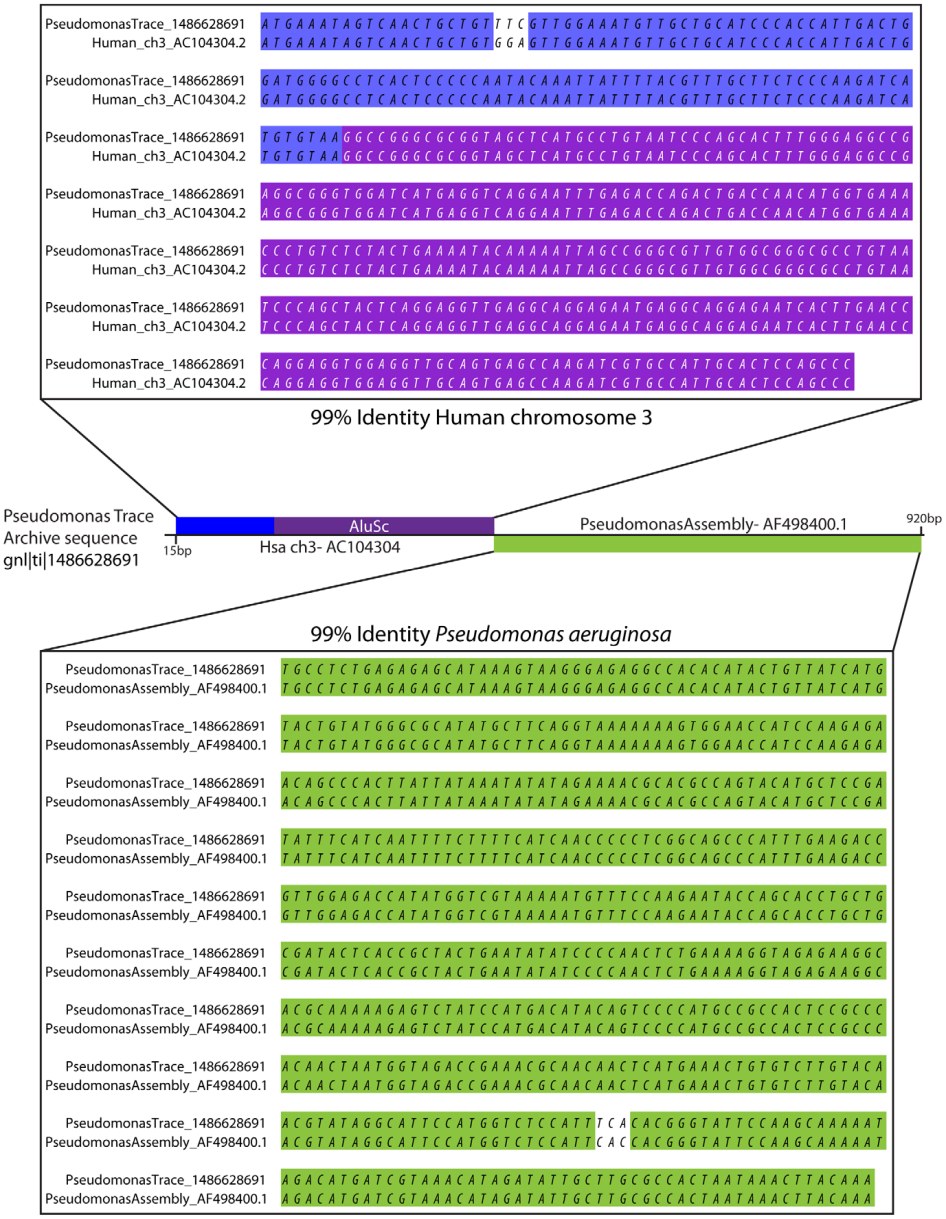
Chimeric NCBI Trace Archive read containing both *Pseudomonas* and human sequence. Approximately one half of this 920 bp sequence entry is >99% identical to human (blue and purple) while the other half is >99% identical to *Pseudomonas aeruginosa* (green). The Alu alignment used to identify this sequence is shown in purple.

Many Alu sequences were found in the Trace archives for species from all phyla (Figure 1A–D, Table S1A–B). Sequence adjacent to captured Alu elements mapped to a unique locus in the human genome, regardless of the species to which the trace entry was derived (Figure 1B). Of the 2,027 non-primate trace archives, 454 (22.39%) were identified as contaminated with human sequence (Table S1B). Notably, a trace sequence collected from the *Pseudomonas aeruginosa WGS* database is chimeric, containing both *Pseudomonas* and human sequence (Figure 2), apparently the result of incorporation of the human contaminant during the cloning process *prior* to sequencing. Interestingly, no contamination was found in any of the 172 influenza virus archives examined.

Ten NCBI genome assemblies were found to contain human sequence (10.64%) (Figures 1C, 1D and Table S1C). Each human sequence found in a genome assembly, regardless of location, is bounded by gaps (N's), indicative of a failure of scaffolding algorithms to integrate these sequences onto chromosomes. Moreoever, the cassava, *Manihot esculenta*, contains contamination in its cDNA sequencing reads, indicating that genome sequence cross contamination is not limited to genome databases. While we were able to identify contamination of human origin in genome assemblies, the relatively higher abundance of trace archive contaminants demonstrates the ability to computationally eliminate these sequences from scaffolds and large contigs.

Over one quarter (28.5% of 42) of UCSC's assemblies were found to contain human sequence (Figure 1C–D and Table S1D). Like NCBI, most contaminants were found in large contigs and scaffolds. One exception is the first 5477 bp of chromosome 11 in zebrafish; this region is 100% identical to human chromosome 4. In addition, the *Xenopus* assembly carries two large blocks of contiguous human sequence (300 kb and 85 kb). A similar number of Ensembl genomes were identified as contaminated (26.8% of 41) while only a few genomes from JGI (1.65% of 545) were identified (Figure 1C–D). JGI's databases have relatively little sequence data (5Mb or less) which may explain this relatively low level of contamination.

## Discussion

The level of contamination found in these databases is significant and worrisome. Trace archive databases are often used in cross species analyses when whole genome sequences are not available or in the analyses of unassembled regions of genomes. With the advent of whole genome re-sequencing and other deep sequencing applications, assemblies are heavily relied upon for data mapping and analyses. Moreover, such contamination potential is a critical consideration when single human sample re-sequencing is performed, as in the case of The Cancer Genome Atlas (www.cancergenome.nih.gov) and the 1,000 Genomes Project ([8]; www.1000genomes.org), as assembly and scaffolding algorithms are unable to distinguish between human sequence and human sequence contamination. This study points to a need for more rigorous pre-sequencing protocols and laboratory standards.

## Methods

Human sequences were identified by screening non-primate databases with the primate specific short interspersed element (SINE) AluY consensus sequence obtained from Repbase [9]. Database screens were performed using the BlastN alignment algorithm [10]. UCSC databases were screened using the BLAT alignment algorithm [11]. Alignments of >80% identity were further evaluated first using Censor [9] to identify any repetitive elements (including AluY). Any non-repetitive sequence was then mapped to NCBI's human assembly using BlastN. Sequences from non-primate databases with >98% identity to human sequence were considered contaminating sequences. The alignment of NCBI trace archive sequences to AluY (Figure 1A) was performed using ClustalW [12] and visualized using Jalview [13]. Non-primate databases screened include the National Center for Biotechnology Information (NCBI) trace archives (2027) and genome assemblies (94) (http://www.ncbi.nlm.nih.gov/), University of California Santa Cruz (UCSC) genome assemblies (42) (http://genome.ucsc.edu/), the Department of Energy's Joint Genome Institute (JGI) blastable DNA databases (545) (http://www.jgi.doe.gov/) and Ensembl's genome assemblies (41) (http://www.ensembl.org/).

## Supporting Information

Table S1 A) NCBI trace archive sequences used for AluY Clustal alignment (Figure 1A). B) Complete list of NCBI non-primate trace archive databases identified as contaminated. C) Sequences in non-primate NCBI genome assemblies identified as human. D) Sequences in non-primate UCSC genome assemblies identified as human.
(XLS)
